# Transgenic overexpression of miR-133a in skeletal muscle

**DOI:** 10.1186/1471-2474-12-115

**Published:** 2011-05-26

**Authors:** Zhongliang Deng, Jian-Fu Chen, Da-Zhi Wang

**Affiliations:** 1 Department of Orthopaedic Surgery, The Second Affiliated Hospital, Chongqing Medical University, Chongqing 400010, P.R.China; 2 McAllister Heart Institute, University of North Carolina, Chapel Hill, NC 27599-7126, USA; 3 Department of Cardiology, Children's Hospital Boston, Harvard Medical School, 320 Longwood Avenue, Boston, MA 02115, USA

**Keywords:** microRNA-133a, skeletal muscle, transgenic, differentiation

## Abstract

**Background:**

MicroRNAs (miRNAs) are a class of non-coding regulatory RNAs of ~22 nucleotides in length. miRNAs regulate gene expression post-transcriptionally, primarily by associating with the 3' untranslated region (UTR) of their regulatory target mRNAs. Recent work has begun to reveal roles for miRNAs in a wide range of biological processes, including cell proliferation, differentiation and apoptosis. Many miRNAs are expressed in cardiac and skeletal muscle, and dysregulated miRNA expression has been correlated with muscle-related disorders. We have previously reported that the expression of muscle-specific miR-1 and miR-133 is induced during skeletal muscle differentiation and miR-1 and miR-133 play central regulatory roles in myoblast proliferation and differentiation in vitro.

**Methods:**

In this study, we measured the expression of miRNAs in the skeletal muscle of mdx mice, an animal model for human muscular dystrophy. We also generated transgenic mice to overexpress miR-133a in skeletal muscle.

**Results:**

We examined the expression of miRNAs in the skeletal muscle of *mdx *mice. We found that the expression of muscle miRNAs, including miR-1a, miR-133a and miR-206, was up-regulated in the skeletal muscle of *mdx *mice. In order to further investigate the function of miR-133a in skeletal muscle in vivo, we have created several independent transgenic founder lines. Surprisingly, skeletal muscle development and function appear to be unaffected in miR-133a transgenic mice.

**Conclusions:**

Our results indicate that miR-133a is dispensable for the normal development and function of skeletal muscle.

## Background

MicroRNAs (miRNAs) are a class of ~ 22 nt non-coding RNAs that regulate gene expression post-transcriptionally [1-3]. The involvement of miRNAs in muscle biology has recently been reported [4-11]. miRNAs regulate the expression of transcription factors and signaling mediators important for cardiac and skeletal muscle development and function [7,12-14]. Aberrant miRNA expression has been observed in muscle diseases, including cardiac and skeletal muscle hypertrophy, heart failure and muscular dystrophy [13,15-17].

A subset of miRNAs, miR-1, miR-133, miR-206 and miR-208, are either specifically or highly expressed in cardiac and skeletal muscle and are called myomiRs [6,7,13]. Among them, miR-133 was shown to promote the proliferation of myoblasts and inhibits their differentiation in cultured skeletal muscle myoblasts. miR-133 enhances myocyte proliferation, at least in part, by reducing protein levels of SRF, a crucial regulator for muscle differentiation [18,19]. miR-133 also inhibits the translation of polypyrimidine tract-binding protein (nPTB), which controls differential transcript splicing during skeletal-muscle differentiation [20]. Paradoxically, miR-1 and miR-133 exert opposing effects to skeletal-muscle development despite originating from the same miRNA polycistronic transcript. Interestingly, miR-1 and miR-133 also produce opposing effects on apoptosis [21]. Additionally, embryonic stem (ES) cell differentiation towards cardiomyocytes is promoted by miR-1 and inhibited by miR-133 [22]. Furthermore, miR-1 and miR-133 are also important regulators of cardiomyocyte differentiation and heart development [22-24].

Primary skeletal-muscle disorders involve different groups of diseases, including muscular dystrophies, inflammatory myopathies and congenital myopathies. Although the number of genes that are involved in muscle disorders increases every year and histological pathology of disease tissue is well documented, the underlying molecular pathways remain poorly understood [25]. Recent studies have begun to link miRNAs to certain muscle-related diseases [6,13,15,26,27]. In a recent report, comprehensive miRNA expression profiling revealed that a total of 185 miRNAs were dysregulated in samples of diseased muscle tissue from 10 different muscle disorders. Five miRNAs (miR-146b, miR-221, miR-155, miR-214 and miR-222) were consistently regulated in almost all samples that were examined [15], suggesting a possible involvement of common miRNA-mediated regulatory mechanisms in muscle disorders. In addition to those studies of miRNA expression on muscle disorders, a direct genetic link has connected miRNA function to muscular hypertrophy [28]. A mutation that is responsible for the exceptional muscularity of Texel sheep has been mapped to a single G-to-A mutation within the 3' UTR of the mRNA encoding myostatin, a member of the transforming growth factor-β (TGFβ) family, which functions to repress muscle growth. This mutation creates a binding site for miR-1 and miR-206, leading to the translational repression of myostatin, which phenocopies the "muscle doubling" that results from the loss of myostatin in mice, cattle, and humans [29,30]. These findings underscore the importance of miRNA-mediated regulation in diverse muscle biological processes and disease status.

In this study, we attempted to determine the function of miR-133 in skeletal muscle. We employed a gain-of-function approach and generated transgenic mice to overexpress miR-133a-1 in skeletal muscle, using the well-characterized muscle creatine kinase (MCK) promoter. Surprisingly, we found that miR-133a-1 transgenic mice appear to be normal. Additional analyses indicated that skeletal muscle development and function were not altered in miR-133a-1 transgenic mice. Our study therefore suggests that miR-133a is dispensable for skeletal muscle development.

## Methods

### Mice

The *mdx *mice which carry a point mutation in the dystrophin gene were obtained from the Jackson Lab [31]. Skeletal muscle was collected from the hind legs of 1 month old *mdx *and control mice for RNA extraction. In order to generate miR-133a-1 transgenic mice, a genomic fragment encoding the precursor and franking sequences of the miR-133a-1 gene, which is located on mouse chromosome 18, was amplified by PCR using mouse genomic DNA as a template. The primers used for amplification are: miR-133a-1F: 5' AAGCTAGCGAATTCCATGTGACCCCTCACACACA 3'; miR-133a-1R: 5' TTCTCGAGACAAGGGGAGCCTGGATCCC 3'. Underlined nucleotide sequences are added adaptors for restriction enzyme digestion. The same primers were used for genotyping of transgenic mice.

The DNA fragment was cloned into a transgenic vector plasmid driving by a muscle-specific muscle creatine kinase (MCK) promoter [32]. The miR-133a-1 transgenic construct was injected into the pronuclei of C57/Bl6 X C3H hybrid embryos and implanted into pseudo-pregnant recipient females by the University of North Carolina Animal Models Core. Five positive founder lines were obtained.

### Ethics Statement

All animal procedures were approved by and performed in accordance with the University of North Carolina Institutional Animal Care and Use Committee under the protocol 08-227.0.

### Northern Blot and RT-PCR Analysis

**Figure 1:**
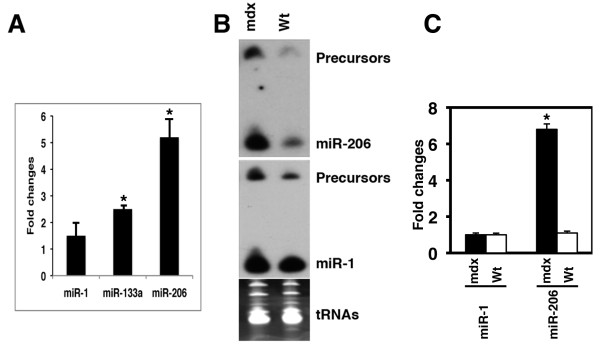
**Increased expression of muscle miRNAs in the skeletal muscle of *mdx *mice**. (A) Fold change of the expression of indicated miRNAs in the skeletal muscle of 1 month old *mdx *mice (n = 3) as detected by qRT-PCR analyses. Statistical differences were determined using the Student's *t*-test; (*) P < 0.05. (B) Northern blot analyses of the expression of miR-1 and miR-206 in the skeletal muscle of *mdx *and the wild type control mice (n = 3). Precursors and mature miRNAs are indicated. tRNAs were used as a loading control. (C) The intensity of mature miRNA products from Northern blot was quantified using the Photoshop Histogram Analysis and was plotted using Microsoft excel and is shown as relative expression level after normalized by tRNA. Statistical differences were determined using the Student's *t*-test; (*) P < 0.05.

**Figure 2:**
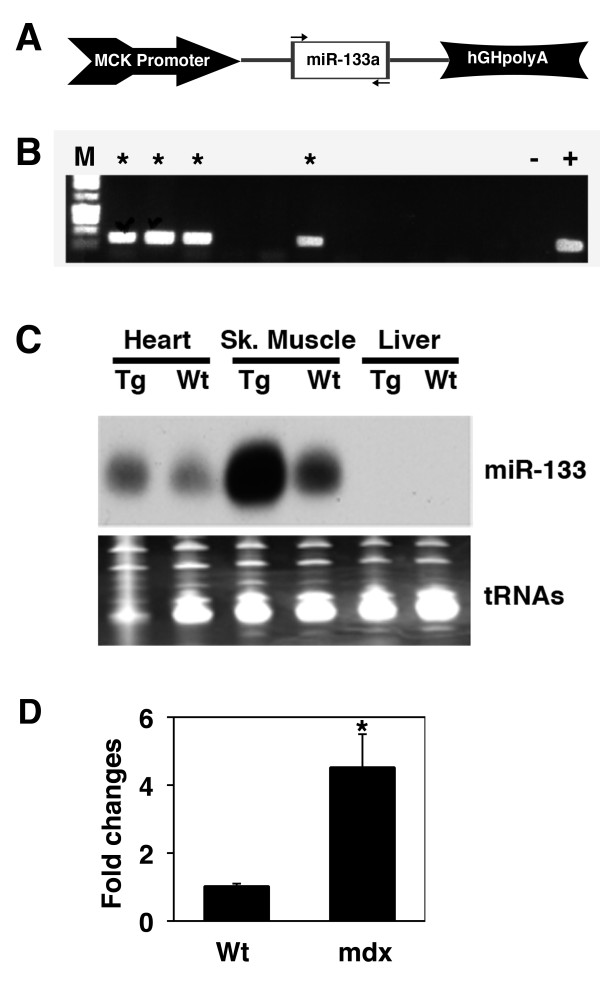
**Generation of miR-133a transgenic mice**. (A) Diagram of transgenic construct used in this study to overexpress miR-133a in the skeletal muscle of transgenic mice. Two arrows represent PCR primers used for genotyping. MCK: muscle creatine kinase; hGH: human growth hormone. (B) PCR genotyping of miR-133a transgenic founder mice. * indicated genotyping positive founder lines; M: DNA size marker; +: Positive control; -: Negative control. (C) Northern blot analyses of miR-133a expression in the heart, skeletal muscle and liver tissues of the wild type and transgenic mice. tRNAs were used as a loading control. (D) The intensity of skeletal muscle expressed miR-133a from Northern blot was quantified using the Photoshop Histogram Analysis and was plotted using Microsoft excel and is shown as relative expression level after normalized by tRNA. Statistical differences were determined using the Student's *t*-test; (*) P < 0.05.

Taqman-based miRNA quantitative RT-PCR (Applied Biosystems) was performed as described [33] using total RNAs isolated from skeletal muscle of 1 month old *mdx *and the control mice (n = 4). MicroRNA Northern blot analyses were performed as described previously [8,18]. Briefly, 20 μg of total RNAs isolated from skeletal muscle of 1 month old *mdx *and the control mice (Figure 1), or from the heart, skeletal muscle and liver tissues of miR-133a-1 transgenic and the control mice (Figure 2), were used and miRNA oligonucleotides with corresponding miRNAs (miR-1a, miR-133a and miR-206) sequences were used as probes. qRT-PCR was repeated three times and Northern blots were performed two times on skeletal muscle of four mice per genotype tested.

### Histological and immunohistochemistry analyses

Histological processing and immunohistochemistry staining of skeletal muscle tissues were performed as described previously [8,34]. Samples were stained with Hematoxylin and Eosin (H&E) for routine examination. Laminin conjugated staining was applied to identify sarcolemmal membranes so that myofiber diameter could be directly visualized. Histological and immunohistochemistry analyses were performed on least 4 animals per genotype and repeated independently. All images were acquired by a camera (UFX-DX; Nikon) mounted on an inverted (TE2000, Nikon) or an upright fluorescence microscope (Microphot-SA; Nikon). Digital fluorescent images were captured at room temperature and the images were processed using SPOT (version 3.5.4 for MacOS; Diagnostic Instruments) software and were scaled down and cropped in Photoshop (Adobe) to prepare the final figures. Gel images were quantified using the Photoshop Histogram Analysis and was plotted using Microsoft excel and is shown as relative expression level after normalized by controls.

## Results

### Changed expression of muscle miRNAs in the skeletal muscle of *mdx *mice

*Mdx *mice harbor a spontaneous mutation in the dystrophin gene [31]. The skeletal muscle of the *mdx *mice is histologically normal early in postnatal development, but starting around 3 weeks muscle necrosis develops with some visible muscle weakness. Therefore, *mdx *mice have been widely used as an animal model to study the skeletal muscle degeneration/regeneration process [35-37]. We examined miRNA expression in the skeletal muscle of *mdx *mice. We found that the expression levels of miR-1, miR-133 and miR-206 were higher in the skeletal muscle of one month-old *mdx *mice (Figure 1A). Northern blot analyses further confirmed a significantly increased expression of miR-206, whereas the expression of miR-1 modestly increased in the muscle muscle of *mdx *mice (Figure 1B, C). These data indicate that these muscle miRNAs might be involved in the regulation of skeletal muscle degeneration and/or regeneration.

### Transgenic overexpression of miR-133a-1 in the skeletal muscle

Recently, miR-133 genes (miR-133a-1 and miR-133a-2) were knocked out from the mouse genome. Analysis of mice that lost either miR-133a-1 or miR-133a-2 revealed that both miRNAs are dispensable for development or viability under normal physiological conditions. However, double knockout mice display defects in the heart [24]. In order to further investigate the function of miR-133 in vivo, we took a gain-of-function approach and generated transgenic mice to overexpress miR-133a-1 in skeletal muscle. We used the well-characterized muscle creatine kinase (MCK) promoter to drive miR-133a-1 expression in skeletal muscle (and to a less extend, cardiac muscle). Genomic DNA from mouse chromosome 18 encoding the miR-133a-1 gene was inserted into an expression vector (Figure 2A). The MCK-miR-133a-1 transgenic construct was injected into fertilized mouse eggs and multiple transgenic founder lines were obtained, as verified by PCR genotyping (Figure 2B). The overexpression of miR-133a-1 in germline-transmitted stable transgenic mice was confirmed by Northern blot analyses. Total RNAs were isolated from indicated tissues, and the overexpression of miR-133a-1 was clearly detected in the skeletal muscle, and to a much less extent, the cardiac muscle, but not in the liver of transgenic mice (Figure 2C, D).

### Normal skeletal muscle development in miR-133 transgenic mice

**Figure 3:**
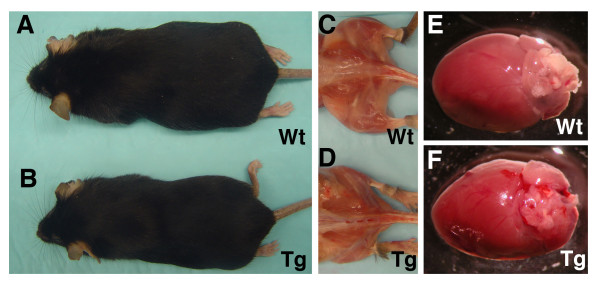
**Normal cardiac and skeletal muscle development in miR-133a transgenic mice**. (A-B) The gross morphology of a 6-month old miR-133a transgenic (Tg) mouse is normal and indistinguishable from its wild type (Wt) littermate (n = 4). (C-D) Representative de-skined mice show normal skeletal muscle morphology in miR-133a transgenic mice (Tg) when compared with the wild type littermate (Wt). (E-F) The heart of miR-133a transgenic mouse (Tg) is indistinguishable from that of the wild type (Wt) littermate.

All miR-133a-1 transgenic mice were viable and fertile without overt abnormality (Figure 3A, B). The body weight and size between wild type and miR-133a-1 transgenic adult mice (ages from 2 to 12 months) were indistinguishable (n = 40, data not shown). There was not difference in skeletal muscle formation or body fat deposit between miR-133a-1 transgenic mice and their littermate controls (Figure 3C, D). Because the MCK promoter also directed miR-133a-1 overexpression in the heart, albeit at lower level, we determined whether heart development was affected in the transgenic mice. However, our results showed that there was no difference in the gross morphology of the adult hearts of miR-133a-1 transgenic mice and wild type controls (Figure 3E, F).

**Figure 4:**
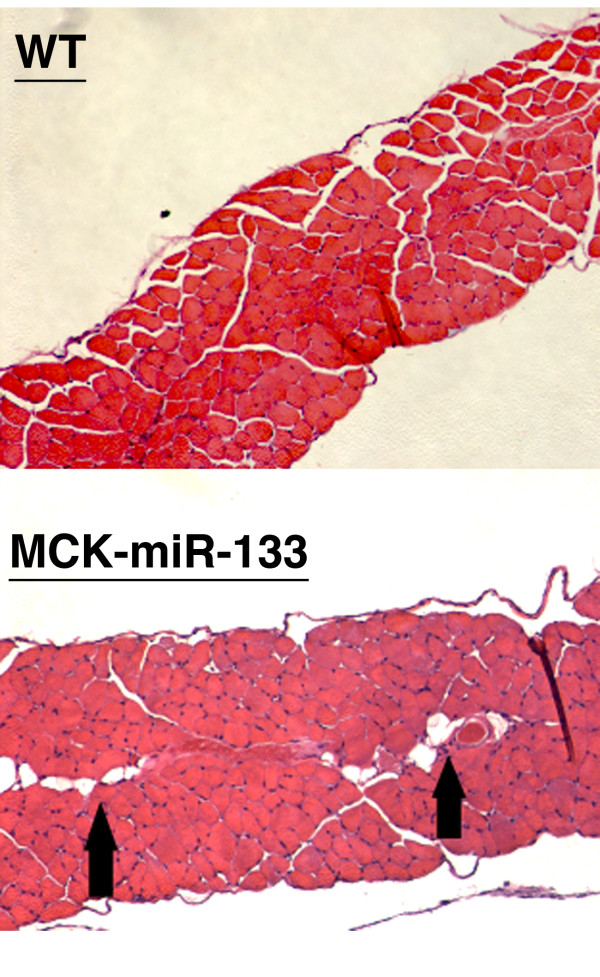
**Histology of skeletal muscle from diaphragm of wild type and miR-133a transgenic mice**. Hematoxylin and eosin (H&E) staining for skeletal muscle tissue sections of diaphragm from 6 month old wild type (Wt) and miR-133a transgenic mice (MCK-miR-133). Black arrows point to vesicles found in the muscle of transgenic mice.

In order to further analyze muscle development, skeletal muscle from the diaphragms of six month old miR-133a-1 transgenic mice was collected and examined by tissue histology (n = 4). As shown in Figure 4, hematoxylin and eosin (H&E) staining of diaphragms indicated that the tissue thickness, muscle cell size and numbers were comparable between miR-133a-1 transgenic mice and the control wild type mice. It is noticeable that there is a slight increase in the vesicles in the transgenic diaphragm (Figure 4, arrows).

**Figure 5:**
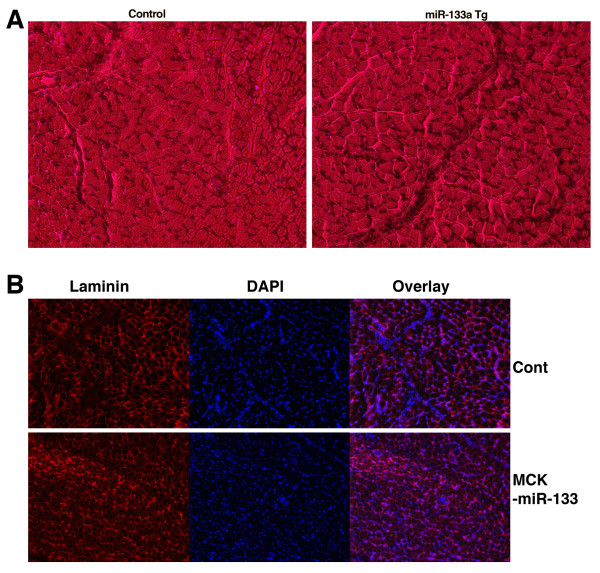
**Histology of skeletal muscle from wild type and miR-133a transgenic mice**. (A) Hematoxylin and eosin (H&E) staining of skeletal muscle tissue transverse sections of EDL (extensor digitorum longus) muscle from 6 month old wild type (Wt) and miR-133a-1 transgenic mice (MCK-miR-133). (B) Immunohistology of skeletal muscle tissue transverse sections of EDL (extensor digitorum longus) muscle from 6 month old wild type (cont) and miR-133a-1 transgenic mice (MCK-miR-133). Laminin staining outlines muscle cells. DAPI staining illustrates position of nuclei.

Similarly, we examined the skeletal muscle of the extensor digitorum longus (EDL) from six month old of both miR-133a-1 transgenic and wild type control mice (n = 4). H&E staining suggested that there was no difference in skeletal muscle morphology between miR-133a-1 transgenic mice and their control littermates (Figure 5A). Immunohistochemistry using an antibody that specifically recognizes laminin, a structural and biologically active component in basement membranes which labels the cell membrane, confirmed that the size and morphology of skeletal muscle in transgenic mice were indistinguishable from that of controls (Figure 5B). Together, these data suggest that miR-133a is dispensable for the normal development and function of skeletal muscle.

## Discussion

In this study, we investigated the function of miR-133a in skeletal muscle. We found that the expression miR-133a, together with that of miR-206 and miR-1a, was induced in the skeletal muscle of *mdx *mice. However, transgenic overexpression of miR-133a-1 in skeletal muscle did not result in a noticeable change in skeletal muscle development and morphogenesis. Our results are consistent with a recent report in which miR-133 loss-of-function mice did not induce overt defects in skeletal muscle [24].

It is known that many miRNAs are tissue-specifically expressed. Among them, miR-1, miR-133, miR-206, miR-208 and miR-499 have been described as muscle specific miRNAs, or myomiRs [6,13]. In addition to their muscle specific expression in normal physiological condition, the expression of some of those myomiRs was shown to be dynamically regulated in pathological and/or diseased muscles. For example, miR-206 levels are elevated in the diaphragm muscle of the *mdx *mouse, an animal model of muscular dystrophy [27]. Furthermore, miR-1 and miR-206 also participate the regulation of skeletal muscle satellite cell proliferation and differentiation [8]. Both gain- and loss-of-function studies have started to uncover the in vivo function of those muscle miRNAs [10,23,34,38-41]. Interestingly, gene targeting of some of the myomiRs demonstrated that they are dispensable for the normal development and function of cardiac and skeletal muscles [10,40]. However, many of those miRNAs are indispensable for stress-responsive muscle remodeling. Recently, we reported that overexpression of miR-208a in the heart, which is normally restricted to cardiac tissue, was sufficient to induce cardiac hypertrophy in transgenic mice [34]. However, in the current study, we did not observe any overt muscle defect in mice expressing the MCK-miR-133a-1 transgene. This may suggest that cardiac and skeletal muscle have adapted distinct requirement for miRNAs for tissue homeostasis.

Given the vast number of miRNAs and the diverse functions in different biological processes observed in the relatively small number of miRNAs studied thus far, it is apparent that many new and unanticipated functions of miRNAs in normal muscle development, function and disorders are waiting to be discovered. Considering that many miRNAs fine-tune gene-expression programs and the intrinsic complexity of miRNA functional models [42,43], it will important in the future to systematically analyze the potential function of miRNAs in both gain- and loss-of-function animal models.

## Conclusions

In this study, we demonstrate that miR-133a is dispensable for the normal development and function of skeletal muscle.

## Competing interests

The authors declare that they have no competing interests.

## Authors' contributions

ZD and DZW designed the experiment; ZD and JFC performed experiments; ZD and DZ. DZW analyzed the data and wrote the manuscript. All authors read and approved the final manuscript.

## Pre-publication history

The pre-publication history for this paper can be accessed here:

http://www.biomedcentral.com/1471-2474/12/115/prepub

## References

[B1] Ambros V microRNAs: tiny regulators with great potential Cell 2001 107 7 823 826 10.1016/S0092-8674(01)00616-X 11779458

[B2] Ambros V The functions of animal microRNAs Nature 2004 431 7006 350 355 10.1038/nature02871 15372042

[B3] Bartel DP MicroRNAs: target recognition and regulatory functions Cell 2009 136 2 215 233 10.1016/j.cell.2009.01.002 19167326 PMC3794896

[B4] Kim HK Lee YS Sivaprasad U Malhotra A Dutta A Muscle-specific microRNA miR-206 promotes muscle differentiation J Cell Biol 2006 174 5 677 687 10.1083/jcb.200603008 16923828 PMC2064311

[B5] Callis TE Deng Z Chen JF Wang DZ Muscling through the microRNA world Exp Biol Med (Maywood) 2008 233 2 131 138 10.3181/0709-MR-237 18222968

[B6] Chen JF Callis TE Wang DZ microRNAs and muscle disorders J Cell Sci 2009 122 Pt 1 13 20 19092056 10.1242/jcs.041723 PMC2714401

[B7] van Rooij E Liu N Olson EN MicroRNAs flex their muscles Trends Genet 2008 24 4 159 166 10.1016/j.tig.2008.01.007 18325627

[B8] Chen JF Tao Y Li J Deng Z Yan Z Xiao X Wang DZ microRNA-1 and microRNA-206 regulate skeletal muscle satellite cell proliferation and differentiation by repressing Pax7 J Cell Biol 190 5 867 879 10.1083/jcb.200911036 PMC2935565 20819939

[B9] Rao PK Kumar RM Farkhondeh M Baskerville S Lodish HF Myogenic factors that regulate expression of muscle-specific microRNAs Proc Natl Acad Sci USA 2006 103 23 8721 8726 10.1073/pnas.0602831103 16731620 PMC1482645

[B10] Williams AH Valdez G Moresi V Qi X McAnally J Elliott JL Bassel-Duby R Sanes JR Olson EN MicroRNA-206 delays ALS progression and promotes regeneration of neuromuscular synapses in mice Science 2009 326 5959 1549 1554 10.1126/science.1181046 20007902 PMC2796560

[B11] Rosenberg MI Georges SA Asawachaicharn A Analau E Tapscott SJ MyoD inhibits Fstl1 and Utrn expression by inducing transcription of miR-206 J Cell Biol 2006 175 1 77 85 10.1083/jcb.200603039 17030984 PMC2064500

[B12] Callis TE Wang DZ Taking microRNAs to heart Trends in molecular medicine 2008 14 6 254 260 10.1016/j.molmed.2008.03.006 18457996

[B13] Williams AH Liu N van Rooij E Olson EN MicroRNA control of muscle development and disease Curr Opin Cell Biol 2009 21 3 461 469 10.1016/j.ceb.2009.01.029 19278845 PMC2692369

[B14] Zhao Y Srivastava D A developmental view of microRNA function Trends Biochem Sci 2007 32 4 189 197 10.1016/j.tibs.2007.02.006 17350266

[B15] Eisenberg I Eran A Nishino I Moggio M Lamperti C Amato AA Lidov HG Kang PB North KN Mitrani-Rosenbaum S Distinctive patterns of microRNA expression in primary muscular disorders Proceedings of the National Academy of Sciences of the United States of America 2007 104 43 17016 17021 10.1073/pnas.0708115104 17942673 PMC2040449

[B16] Tatsuguchi M Seok HY Callis TE Thomson JM Chen JF Newman M Rojas M Hammond SM Wang DZ Expression of microRNAs is dynamically regulated during cardiomyocyte hypertrophy Journal of molecular and cellular cardiology 2007 42 6 1137 1141 10.1016/j.yjmcc.2007.04.004 17498736 PMC1934409

[B17] Thum T Galuppo P Wolf C Fiedler J Kneitz S van Laake LW Doevendans PA Mummery CL Borlak J Haverich A MicroRNAs in the human heart: a clue to fetal gene reprogramming in heart failure Circulation 2007 116 3 258 267 10.1161/CIRCULATIONAHA.107.687947 17606841

[B18] Chen JF Mandel EM Thomson JM Wu Q Callis TE Hammond SM Conlon FL Wang DZ The role of microRNA-1 and microRNA-133 in skeletal muscle proliferation and differentiation Nat Genet 2006 38 2 228 233 10.1038/ng1725 16380711 PMC2538576

[B19] Niu Z Li A Zhang SX Schwartz RJ Serum response factor micromanaging cardiogenesis Current opinion in cell biology 2007 19 6 618 627 10.1016/j.ceb.2007.09.013 18023168 PMC2735128

[B20] Boutz PL Chawla G Stoilov P Black DL MicroRNAs regulate the expression of the alternative splicing factor nPTB during muscle development Genes & development 2007 21 1 71 84 10.1101/gad.1500707 17210790 PMC1759902

[B21] Xu C Lu Y Pan Z Chu W Luo X Lin H Xiao J Shan H Wang Z Yang B The muscle-specific microRNAs miR-1 and miR-133 produce opposing effects on apoptosis by targeting HSP60, HSP70 and caspase-9 in cardiomyocytes Journal of cell science 2007 120 Pt 17 3045 3052 17715156 10.1242/jcs.010728

[B22] Ivey KN Muth A Arnold J King FW Yeh RF Fish JE Hsiao EC Schwartz RJ Conklin BR Bernstein HS MicroRNA regulation of cell lineages in mouse and human embryonic stem cells Cell stem cell 2008 2 3 219 229 10.1016/j.stem.2008.01.016 18371447 PMC2293325

[B23] Zhao Y Ransom JF Li A Vedantham V von M Muth AN Tsuchihashi T McManus MT Schwartz RJ Srivastava D Dysregulation of cardiogenesis, cardiac conduction, and cell cycle in mice lacking miRNA-1-2 Cell 2007 129 2 303 317 10.1016/j.cell.2007.03.030 17397913

[B24] Liu N Bezprozvannaya S Williams AH Qi X Richardson JA Bassel-Duby R Olson EN microRNA-133a regulates cardiomyocyte proliferation and suppresses smooth muscle gene expression in the heart Genes Dev 2008 22 23 3242 3254 10.1101/gad.1738708 19015276 PMC2600761

[B25] Davies KE Nowak KJ Molecular mechanisms of muscular dystrophies: old and new players Nature reviews 2006 7 10 762 773 10.1038/nrm2024 16971897

[B26] McCarthy JJ Esser KA MicroRNA-1 and microRNA-133a expression are decreased during skeletal muscle hypertrophy J Appl Physiol 2007 102 1 306 313 17008435 10.1152/japplphysiol.00932.2006

[B27] McCarthy JJ Esser KA Andrade FH MicroRNA-206 is overexpressed in the diaphragm but not the hindlimb muscle of mdx mouse American journal of physiology 2007 293 1 C451 457 10.1152/ajpcell.00077.2007 17459947

[B28] Clop A Marcq F Takeda H Pirottin D Tordoir X Bibe B Bouix J Caiment F Elsen JM Eychenne F A mutation creating a potential illegitimate microRNA target site in the myostatin gene affects muscularity in sheep Nature genetics 2006 38 7 813 818 10.1038/ng1810 16751773

[B29] Tobin JF Celeste AJ Myostatin, a negative regulator of muscle mass: implications for muscle degenerative diseases Current opinion in pharmacology 2005 5 3 328 332 10.1016/j.coph.2005.01.011 15907921

[B30] Lee SJ Regulation of muscle mass by myostatin Annual review of cell and developmental biology 2004 20 61 86 10.1146/annurev.cellbio.20.012103.135836 15473835

[B31] Sicinski P Geng Y Ryder-Cook AS Barnard EA Darlison MG Barnard PJ The molecular basis of muscular dystrophy in the mdx mouse: a point mutation Science 1989 244 4912 1578 1580 10.1126/science.2662404 2662404

[B32] Johnson JE Wold BJ Hauschka SD Muscle creatine kinase sequence elements regulating skeletal and cardiac muscle expression in transgenic mice Mol Cell Biol 1989 9 8 3393 3399 2796990 10.1128/mcb.9.8.3393-3399.1989 PMC362385

[B33] Huang ZP Chen JF Regan JN Maguire CT Tang RH Dong XR Majesky MW Wang DZ Loss of microRNAs in neural crest leads to cardiovascular syndromes resembling human congenital heart defects Arterioscler Thromb Vasc Biol 30 12 2575 2586 10.1161/ATVBAHA.110.213306 PMC2988089 20884876

[B34] Callis TE Pandya K Seok HY Tang RH Tatsuguchi M Huang ZP Chen JF Deng Z Gunn B Shumate J MicroRNA-208a is a regulator of cardiac hypertrophy and conduction in mice J Clin Invest 2009 119 9 2772 2786 10.1172/JCI36154 19726871 PMC2735902

[B35] De la Porte S Morin S Koenig J Characteristics of skeletal muscle in mdx mutant mice Int Rev Cytol 1999 191 99 148 10343393 10.1016/s0074-7696(08)60158-8

[B36] Messina S Mazzeo A Bitto A Aguennouz M Migliorato A De Pasquale MG Minutoli L Altavilla D Zentilin L Giacca M VEGF overexpression via adeno-associated virus gene transfer promotes skeletal muscle regeneration and enhances muscle function in mdx mice FASEB J 2007 21 13 3737 3746 10.1096/fj.07-8459com 17575261

[B37] Greco S De Simone M Colussi C Zaccagnini G Fasanaro P Pescatori M Cardani R Perbellini R Isaia E Sale P Common micro-RNA signature in skeletal muscle damage and regeneration induced by Duchenne muscular dystrophy and acute ischemia FASEB J 2009 23 10 3335 3346 10.1096/fj.08-128579 19528256

[B38] Zhao Y Samal E Srivastava D Serum response factor regulates a muscle-specific microRNA that targets Hand2 during cardiogenesis Nature 2005 436 7048 214 220 10.1038/nature03817 15951802

[B39] Olson EN Gene regulatory networks in the evolution and development of the heart Science 2006 313 5795 1922 1927 10.1126/science.1132292 17008524 PMC4459601

[B40] van Rooij E Sutherland LB Qi X Richardson JA Hill J Olson EN Control of stress-dependent cardiac growth and gene expression by a microRNA Science 2007 316 5824 575 579 10.1126/science.1139089 17379774

[B41] van Rooij E Quiat D Johnson BA Sutherland LB Qi X Richardson JA Kelm RJ Jr Olson EN A family of microRNAs encoded by myosin genes governs myosin expression and muscle performance Dev Cell 2009 17 5 662 673 10.1016/j.devcel.2009.10.013 19922871 PMC2796371

[B42] Ambros V The evolution of our thinking about microRNAs Nat Med 2008 14 10 1036 1040 10.1038/nm1008-1036 18841144

[B43] Baek D Villen J Shin C Camargo FD Gygi SP Bartel DP The impact of microRNAs on protein output Nature 2008 455 7209 64 71 10.1038/nature07242 18668037 PMC2745094

